# Highly Efficient Separation of Ethanol Amines and Cyanides via Ionic Magnetic Mesoporous Nanomaterials

**DOI:** 10.3390/ijms25126470

**Published:** 2024-06-12

**Authors:** Yuxin Zhao, Fangchao Yang, Jina Wu, Gang Qu, Yuntao Yang, Yang Yang, Xiaosen Li

**Affiliations:** 1 State Key Laboratory of NBC Protection for Civilian, Beijing 102205, China; zyx161202@163.com (Y.Z.); wujina09@163.com (J.W.); qu0612@126.com (G.Q.); 18210313698@139.com (Y.Y.); 2 School of Chemistry and Chemical Engineering, Nanjing University of Science & Technology, Nanjing 210094, China; 18861984587@163.com

**Keywords:** chemical warfare agents, ethanol amines, cyanide, ionic magnetic mesoporous materials, adsorption

## Abstract

Simple and efficient sample pretreatment methods are important for analysis and detection of chemical warfare agents (CWAs) in environmental and biological samples. Despite many commercial materials or reagents that have been already applied in sample preparation, such as SPE columns, few materials with specificity have been utilized for purification or enrichment. In this study, ionic magnetic mesoporous nanomaterials such as poly(4-VB)@M-MSNs (magnetic mesoporous silicon nanoparticles modified by 4-vinyl benzene sulfonic acid) and Co^2+^@M-MSNs (magnetic mesoporous silicon nanoparticles modified by cobalt ions) with high absorptivity for ethanol amines (EAs, nitrogen mustard degradation products) and cyanide were successfully synthesized. The special nanomaterials were obtained by modification of magnetic mesoporous particles prepared based on co-precipitation using -SO_3_H and Co^2+^. The materials were fully characterized in terms of their composition and structure. The results indicated that poly(4-VB)@M-MSNs or Co^2+^@M-MSNs had an unambiguous core-shell structure with a BET of 341.7 m^2^·g^−1^ and a saturation magnetization intensity of 60.66 emu·g^−1^ which indicated the good thermal stability. Poly(4-VB)@M-MSNs showed selective adsorption for EAs while the Co^2+^@M-MSNs were for cyanide, respectively. The adsorption capacity quickly reached the adsorption equilibrium within the 90 s. The saturated adsorption amounts were MDEA = 35.83 mg·g^−1^, EDEA = 35.00 mg·g^−1^, TEA = 17.90 mg·g^−1^ and CN^−^= 31.48 mg·g^−1^, respectively. Meanwhile, the adsorption capacities could be maintained at 50–70% after three adsorption–desorption cycles. The adsorption isotherms were confirmed as the Langmuir equation and the Freundlich equation, respectively, and the adsorption mechanism was determined by DFT calculation. The adsorbents were applied for enrichment of targets in actual samples, which showed great potential for the verification of chemical weapons and the destruction of toxic chemicals.

## 1. Introduction

Vesicants and systemic toxicants represent two types of highly hazardous organic compounds that are also identified as lethal chemical warfare agents (CWAs, Table S1 as described below). The production, storage, and deployment of those were strictly prohibited after the Chemical Weapons Convention (CWC) [1,2]. Despite the strict regulations, incidents of terrorist attacks employing chemical weapons have, unfortunately, been confirmed in recent years. Vesicant compounds usually include sulfur mustards (SMs), nitrogen mustards (NMs), and lewisite (L1, L2, and L3). In contrast to SMs, which have been fully investigated, NMs such as bis(2-chloroethyl)ethylamine (HN1), bis(2-chloroethyl)methylamine (HN2), and tris(2-chloroethyl)amine (HN3) have attracted increasing attention in the last few years. Notably, NMs are facile to synthesize, stable, and challenging to counteract [3,4]. The toxicity of NMs primarily destroys the skin, eyes, and respiratory tract. Similar to SMs, NMs generate mucosal damage and cellular dysfunction through alkylation reactions, leading to a cascade of detrimental effects on biological molecules such as DNA and proteins and even death [5,6]. Hence, the development of a convenient and sensitive detection method for NMs both in the environment and in biological samples is of great importance for the verification of chemical weapons and for public safety.

**Figure 1:**
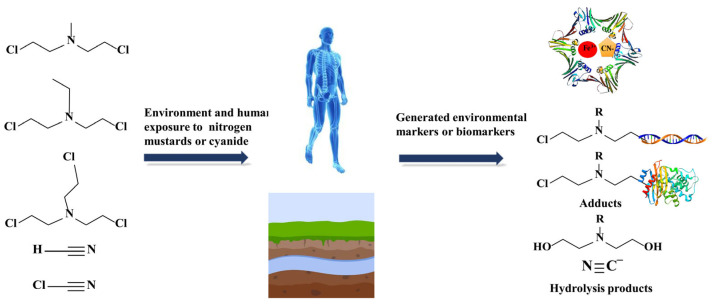
The forms of nitrogen mustards and cyanide present in the environment and in humans.

Ethanol amines (EAs) are recognized as the markers for the detection of NMs in the environment and biological samples (seen in Figure 1); they are generated by the replacement of chlorine atoms by hydroxy groups [7,8]. In contrast to SMs, for which numerous analytical methods had been established [9,10,11], few detection technologies for NMs have been employed. Liquid chromatography-mass spectrometry (LC-MS) or gas chromatography-mass spectrometry (GC-MS) techniques have been utilized for the detection of EAs [7,12,13]. Due to the volatility and polarity of EAs, additional derivatizations such as trimethylsilyl derivatization by BSTFA (N, O-bis(trimethylsilyl trifluoroacetamide)) [14] or fluoro derivatization by HFBI (heptafluorobutyryl imidazole) [8,15] are essential for GC-MS analysis. However, impurities such as inorganic salts, amines, or even proteins in the samples are harmful to the derivatization process. In particular, the low purification efficiency is especially serious for LC-MS analysis. Consequently, highly efficient sample pretreatment can be recognized as the primary determinant of EA analysis and detection. Traditional approaches usually depend on liquid-liquid extraction (LLE) or solid-phase extraction (SPE). Unfortunately, LLE methods exhibit low recovery and limited specificity towards the target analyte [16,17]. Appropriate SPE processes can efficiently enrich the compounds [18,19]. However, commercial SPE columns usually lack active sites for the specified targets, which might reduce the separation rate and diminish reusability or specificity. Furthermore, variability in particle size and homogeneity across different commercial columns limits their application. Hence, the development of specific adsorbent materials for the separation and enrichment of EAs in complex matrices is of particular significance for the trace detection of NMs.

The other highly toxic compounds used as systemic toxic agents are cyanogen compounds, including hydrocyanic acid and cyanogen chloride, which are both identified as CWAs. Cyanide has been widely utilized as a complexometric reagent or a chemical precursor in dyeing [20], electroplating [21], metallurgy [22,23], pharmaceuticals [24], and synthesis of pesticides [25]. Due to its high toxicity, severe poisoning and acute death would occur even with minute exposures via skin contact, inhalation, or ingestion. After the accidental exposure of the human body to cyanide, it can rapidly generate CN^–^ (seen in Figure 1), which exhibits strong chelating properties. This particular ion can tightly bind to Fe^3+^ in mitochondrial oxidases of iron-rich cells, impairing cytochrome oxidase activity and destroying oxidation-reduction processes. Cellular asphyxiation and tissue hypoxia ensue, and ultimate death will occur immediately without urgent medical treatment [26,27].

As a class of toxic and hazardous chemicals widely applied in industry, multiple detection methods have been established for cyanide, such as spectrophotometry [28], colorimetry [29], electrochemical methods [30], chromatography [31], and chromatography-mass spectrometry [32]. However, with the widespread utilization of cyanide compounds, effective removal measures should also be addressed. Three primary treatments are employed for the decontamination of cyanide in wastewater and solid waste, but problems still exist. Direct decomposition approaches such as alkaline chlorination oxidation are not beneficial for metal complex cyanides and increase the total solid content of water [23,33,34]. Acidification and ion exchange inadvertently generate the highly toxic compound HCN during processing [35,36]. In the last few years, cyanide removal by the adsorption process has come to be regarded as a safe and efficient management measure, implemented with activated carbon or resins. Notably, nanomaterials have been applied for adsorption, but they are only suitable for the removal of low concentrations of cyanide, with a low capacity of 0.14 mg·g^−1^ due to limited active sites or low reusability [37,38,39,40]. In addition, the synthesis of adsorbent materials might be time-consuming or costly. Therefore, the development of convenient and cost-effective adsorption materials with high adsorption capacity is significantly important for production processes using cyanide and for safe disposal afterward.

**Scheme 1:**
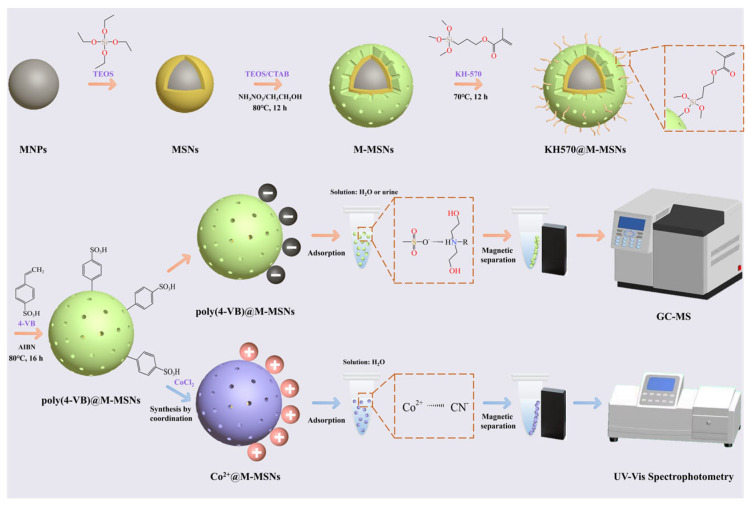
The schematic diagram of the synthesis and application of the poly(4-VB)@M-MSNs and Co^2+^@M-MSNs.

In recent years, magnetic mesoporous materials with expansive specific surface area and excellent magnetic separation capabilities have been widely applied to the adsorption process [41,42,43,44,45]. The use of Fe_3_O_4_ as the magnetic core can effectively obviate time-consuming steps such as centrifugation or filtration [46]. Additionally, the capability for fast separation and the stable structure of the mesoporous silica layer above the magnetic core provides more modifiable sites [47,48]. In this study, novel adsorbent nanomaterials, both for the enrichment and purification of EAs from complex biological samples and for the efficient removal of cyanide, were synthesized in modified layers combined with a magnetic core. The preparation and application procedure is shown in Scheme 1. The polymerization of 4-vinyl benzene sulfonic acid (4-VB) and the complexation with cobalt ions provided the modified groups. Two kinds of ionic magnetic mesoporous nanomaterials were obtained: poly(4-VB)@M-MSNs and Co^2+^@M-MSNs, respectively. The synthesized materials showed excellent adsorption properties. The materials were characterized by several techniques to confirm the composition and structure. Most importantly, the materials were successfully applied to chemical weapons exposure samples for the detection of NMs and removal of cyanide, respectively. The results were confirmed to be correct by the Organization for the Prohibition of Chemical Weapons (OPCW).

## 2. Results

### Characterization

**Figure 2:**
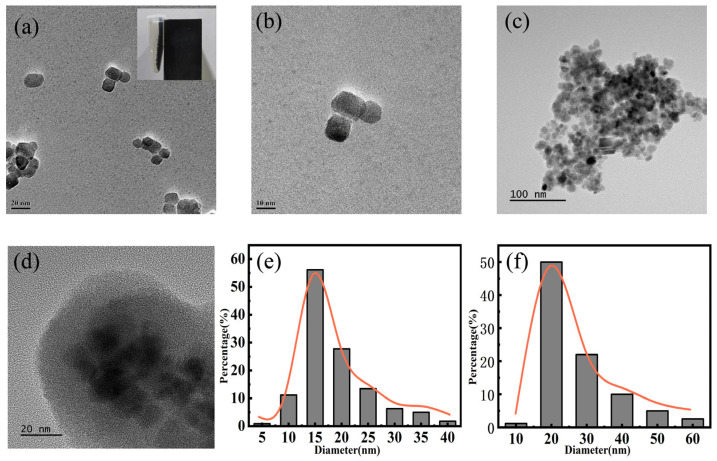
SEM images of Fe_3_O_4_ (**a**,**b**) and TEM images of M-MSNs (**c**,**d**); (**e**) Particle size distribution of Fe_3_O_4_; (**f**) Particle size distribution of M-MSNs.

Figure 2a demonstrates the synthetic nanomaterials with strong magnetic properties. Field emission scanning electron microscopy (SEM) and transmission electron microscopy (TEM) were been utilized to characterize the morphology of the materials. Figure 2a,b shows the SEM images of Fe_3_O_4_. The particle size of the products is approximately 15 nm, with a particle size distribution as illustrated in Figure 2e, which is consistent with the calculated results of the XRD spectrum (Table S2). No obvious agglomeration was observed, which indicated the good dispersion and excellent magnetic properties of Fe_3_O_4._ In the upper right corner of Figure 2a, an external magnet was applied to attract Fe_3_O_4_. Figure 2c,d shows the TEM images of M-MSNs, in which the dark part was Fe_3_O_4_ and the light part was due to the SiO_2_ layer, with dimensions of approximately 20 nm. The particle size distribution of M-MSNs is shown in Figure 2f (calculated results of XRD spectra are shown in Table S3). The thickness of the SiO_2_ layer was about 2.5 nm. The results of electron microscopy indicated that the Fe_3_O_4_ as the core was successfully modified with SiO_2_. The core-shell structure of the material was obvious, with good dispersion performance in the water. Meanwhile, successful coating with the SiO_2_ layer reduced the influence of environmental or biological impurities and improved the stability of Fe_3_O_4_ particles for adsorption.

**Figure 3:**
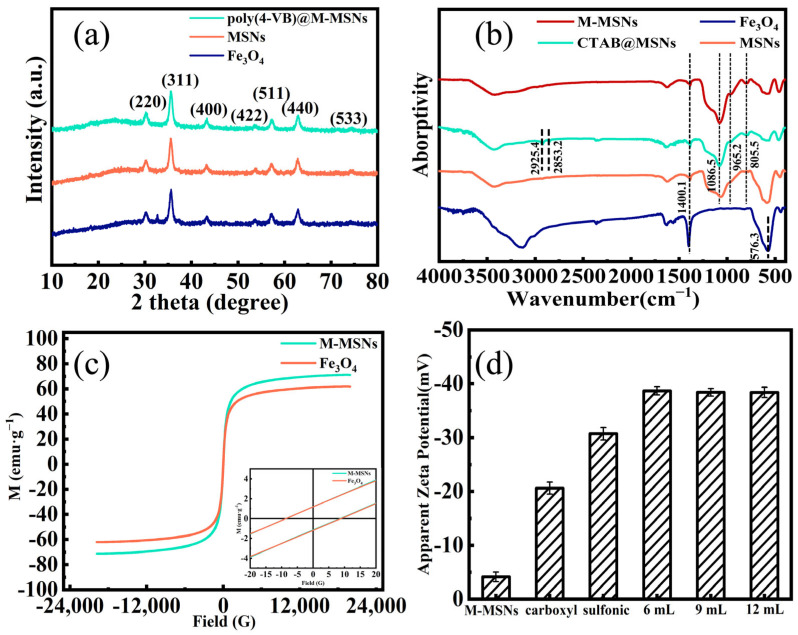
(**a**) XRD of Fe_3_O_4_, MSNs, and poly(4-VB)@M-MSNs; (**b**) FT-IR of Fe_3_O_4_, MSNs, CTAB@MSNs, and M-MSNs; (**c**) VSM of Fe_3_O_4_ and M-MSNs; (**d**) Zeta potential under different conditions.

The crystal structure and crystallinity of Fe_3_O_4_, MSNs, and poly(4-VB)@M-MSNs were fully examined through X-ray diffraction (XRD) analysis. The degree of crystallinity observed in the XRD spectra reflected the intensity and clarity of the diffraction peaks. The results are shown in Figure 3a; distinct and well-defined diffraction peaks were observed at 2θ positions of 30.07°, 35.52°, 43.23°, 53.65°, 57.12°, 62.74°, and 74.10°. Compared to the Fe_3_O_4_ standard card (JCPDS 79-0419), these peaks correspond to the (220), (311), (400), (422), (511), (440), and (533) lattice planes of Fe_3_O_4_ crystals, respectively [49]. The results also confirmed the successful synthesis of Fe_3_O_4_ with the anti-spinel structure. Notably, the absence of diffraction peaks corresponding to other impurities in the spectra demonstrated the purity of the synthesized product. Furthermore, the flowing modifications including SiO_2_ coating and sulfonic acid functionalization did not introduce any significant alterations. The results suggested that neither the ultrasonic treatment nor the incorporation of functionalized reagents during synthesis resulted in phase changes in the Fe_3_O_4_ core. The materials maintained their magnetic separation properties, which was beneficial for the adsorbent separation of EAs and cyanide.

The molecular structure and chemical bonding evolution of Fe_3_O_4_ (Figure 3b), both pre- and post-modification, were determined by Fourier-transform infrared spectrometry (FT-IR). The vibrational absorption peak observed at 576 cm^−1^ corresponded to the Fe-O bond within Fe_3_O_4_. Due to modification with SiO_2_ and the following alterations, the special peak of 576 cm^−1^ was split, which was attributed to the reduction of Fe_3_O_4_ particle size to the nanoscale. Due to its nano-size, the bond force constant on the particle surface increases, leading to a large number of atoms being exposed at the surface and causing nonlocal electron rearrangement. This suggests that the synthesized Fe_3_O_4_ is a prototypical nanostructure. Meanwhile, the rearrangement of nonlocal electrons indicated the characteristic nanostructure of the synthesized Fe_3_O_4_. Additional absorption peaks at 1080, 965, and 805 cm^−1^ were recognized as the antisymmetric and symmetric stretching vibrations of Si-O-Si, as well as interlayer Si-OH bending vibrations and Si-O-Si bending vibrations, respectively. The results confirmed successful coating with the SiO_2_ layer, which was consistent with the TEM results. In particular, the C-H vibrational absorption peaks attributed to the templating agent CTAB at 2925 and 2853 cm^−1^ could be easily observed, and they also disappeared after treatment with ammonium nitrate/ethanol. This removal of the peaks indicated the sufficient removal of the CTAB template and the successful synthesis of mesoporous silica. Hence, the structure of the mesoporous silica could increase the adsorption sites and the adsorption capacity for EAs and cyanide targets.

To investigate the magnetic characteristics of the synthesized Fe_3_O_4_ nanoparticles and the SiO_2_-modified nanoparticles (M-MSNs), a vibrating sample magnetometer (VSM) was utilized. As shown in Figure 3c, the hysteresis curves of these materials were tested at room temperature. Both materials exhibited negligible residual coercivity and remanent magnetization, which indicated a superparamagnetic nature devoid of hysteresis. When the applied magnetic field was canceled, the magnetism vanished, and no inherent magnetic retention could be observed. Additionally, the magnetization intensity was consistent with the direction of the applied magnetic field. The saturation magnetization intensity of Fe_3_O_4_ was 71.17 emu·g^−1^, while that of M-MSNs was slightly reduced to 60.66 emu·g^−1^ due to the SiO_2_ layer coating. Above all, M-MSNs still exhibited robust magnetic response, which ensured effective magnetic separation within 5 min. The results indicate that the coating of mesoporous SiO_2_ had little effect on the magnetic properties of the Fe_3_O_4_ core. Consequently, the magnetic responsiveness maintained by the M-MSNs gave the nanomaterials effective magnetic separation ability.

The surface potential of the magnetic nanomaterials before and after the modification was evaluated with a zeta potential meter. On account of the electrostatic interactions between the targets and the synthesized nanomaterials, the quantity of ion charge around the surface of the materials could influence its adsorption capacity. The impact of sulfonic acid (-SO_3_H) and carboxylic acid (-COOH) groups, as well as the dosage of the silylated coupling agent and KH-570, on the negative charge on the surface were fully investigated. As the oxygenated acidic moieties, the acidity of the -SO_3_H and -COOH groups was due to the protonation. The electrostatic interaction strength between the functional groups and the mesoporous silica could be measured by the electric potential.

The Zeta potential of the material was characterized (Figure S6). As illustrated in Figure 3d, the original M-MSNs exhibited a zeta potential of around −4.14 mV. After modification with -COOH or -SO_3_H, the potential was significantly decreased, indicating the successful loading of these functional groups onto the M-MSNs surface and the generation of a negative charge. -SO_3_H-modified poly(4-VB)@M-MSNs displayed a more negative zeta potential (−29.8 mV) than -COOH-modified COOH@M-MSNs (−20.2 mV). The results proved that -SO_3_H could enhance the negative charge density and improve the target adsorption capacity compared to -COOH. Consequently, -SO_3_H was selected for further optimization. Given that -SO_3_H modification occurred via a polymerization reaction with KH-570 double bonds, the dosage of KH-570 was optimized. KH-570-grafted mesoporous silica was used to react with -SO_3_H in the amounts of 3 mL, 6 mL, 9 mL, and 12 mL, respectively. As shown in Figure 3d, the dosage of 6 mL corresponded to an electric potential of −38.7 mV, which no longer increased even though the dosage of KH-570 improved.

**Figure 4:**
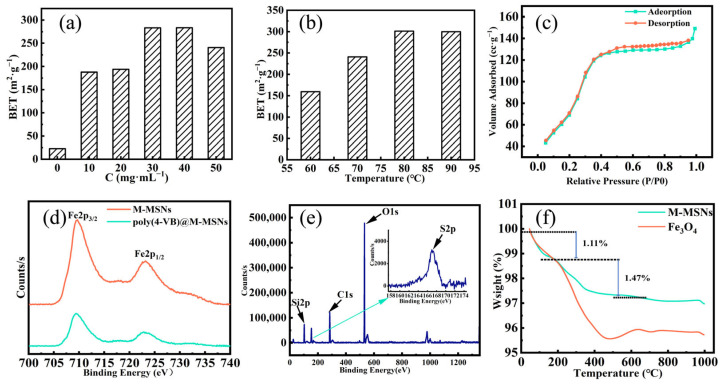
(**a**) Effect of ammonium nitrate concentration on BET; (**b**) Effect of template removal temperature on BET; (**c**) N_2_ adsorption-desorption isotherm plots for magnetic mesoporous materials; (**d**) XPS spectra of Fe 2p; (**e**) XPS spectra of poly(4-VB)@M-MSNs; (**f**) TGA of Fe_3_O_4_ and M-MSNs.

Nitrogen adsorption–desorption analyses were applied to investigate the internal pore architecture of M-MSNs. The synthesis of the magnetic mesoporous silica materials involved the formation of CTAB template micelles, hydrolysis of a silica source, and removal of subsequent templates. Accordingly, the influence of the concentration of ammonium nitrate and temperature on the template removal efficiency was investigated. As depicted in Figure 4a, the concentration of ammonium nitrate/ethanol solution utilized for the removal of the CTAB template was optimized from 0 to 50 mg·mL^−1^. The BET surface area increased to 283.3 m^2^·g^−1^ with the concentration reaching up to 30 mg·mL^−1^ (Figure S1). Further improvement in ammonium nitrate concentration did not increase the BET surface area. Hence, for subsequent synthesis of the materials, 30 mg·mL^−1^ of ammonium nitrate was applied. Subsequently, the reaction temperature was optimized from 60 to 90 °C. As indicated in Figure 4b, the BET surface area reached 301.3 m^2^·g^−1^ when the temperature was up to 80°C (Figure S2). However, further improvement of the temperature did not induce any significant change, indicating that CTAB removal was sufficient at 80 °C with a 30 mg·mL^−1^ ammonium nitrate concentration. Figure 4c presents the nitrogen adsorption–desorption isotherms of the synthesized M-MSNs. Notably, in the relative pressure range of 0.45–0.98, a discernible hysteresis loop characteristic of a type IV isotherm along with a uniform pore size distribution could be observed. The specific surface area of M-MSNs was determined to be 341.7 m^2^·g^−1^, with an average pore size of 2.700 nm (Figure S3). The above results revealed the substantial specific surface area and mesopore volume of M-MSNs, which had excellent target-adsorption capabilities.

To investigate the composition, X-ray photoelectron spectroscopy (XPS) was applied (Tables S4–S6 and Figures S4 and S7). Figure 4d shows the Fe2p XPS spectra of the poly(4-VB)@M-MSN materials. Before synthesis, significant peaks at binding energies of 710.08 and 724.08 eV corresponded to the characteristic peaks of Fe2p_3/2_ and Fe2p_1/2_ for Fe^3+^ of Fe_2_O_3_ and Fe^2+^ of FeO, respectively. The visible peaks at binding energies of 710.48 and 724.08 eV indicated that the Fe_3_O_4_ magnetic nanocore maintained the structure during the synthesis, consistent with previous characterizations. In Figure 4e, the characteristic peak of Si2p emerged at 102.58 eV confirming the presence of SiO_2_ in the poly(4-VB)@M-MSN composite. After modification with the -SO_3_H, the characteristic peak of S2p appeared at 167.38 eV, indicating the successful synthesis of the poly(4-VB)@M-MSN material.

The thermogravimetric analysis (TGA) curves of Fe_3_O_4_ and M-MSN materials, as presented in Figure 4f, reflected the stability of magnetic nanomaterials at different temperatures. The initial weight loss was at 120 °C with a mass loss of 1.107%, which was attributed to the evaporation of solvent adsorbed on the surface or the evaporation of free water. Then, the weight loss of 1.567% in the range of 200–600 °C indicated the thermal decomposition of the mesoporous SiO_2_ layer and the evaporation of crystallization water. Due to the stability of Fe_3_O_4_, the residual rate showed no change when the temperature was above 600 °C. The results confirmed the favorable thermal stability exhibited by the magnetic nanomaterials derived from Fe_3_O_4_.

## 3. Discussion

### 3.1. Optimum pH of Cyanide Adsorption

Cyanide can chelate metal ions, and the chelated cyanide–metal complexes have different absorption wavelengths. Therefore, the qualitative and quantitative analysis of cyanide could be carried out by measuring the absorbance value of cyanide–metal complexes. Different metal ions such as Ca^2+^, Co^2+^, Cu^2+^, Fe^2+,^ and Mg^2+^ with the chelation reactions to cyanide were fully investigated. Finally, Co^2+^ was selected as the probe ion for qualitative and quantitative detection of cyanide. The content of cyanide in the solution was determined by measuring the absorbance values of cyanide–cobalt chelates.

**Figure 5:**
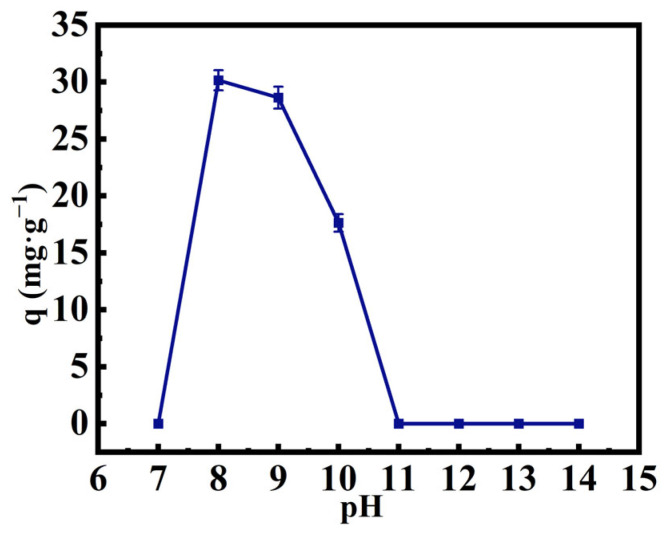
Effect of pH on the adsorption of cyanide.

CN^−^ easily underwent hydrolysis and produced volatile hydrogen cyanide (HCN) if the pH was acidic. Thus, the adsorption behavior of CN^−^ by magnetic nanomaterials was revealed with a pH range of 7–14. We accurately measured the cyanide solution with a molar concentration of 10 mmol·L^−1^ 0.3 mL, diluted it with deionized water to 3 mL, and adjusted it with NaOH solution to make pH = 7–14. Subsequently, 3.0 mg of Co^2+^@M-MSN materials were introduced, followed by adsorption for 10 min at 26 °C. The supernatant was obtained by magnetic separation, and the content of the CN^−^ was detected via the spectrophotometric method. As shown in Figure 5, the adsorbent exhibited negligible capacity for CN^−^ absorption when pH = 7. The phenomenon could be attributed to competition by the proton (H^+^) ions with Co^2+^ for CN^−^ binding sites on the adsorbent. Notably, the adsorption capacity reached 30.02 mg·g^−1^ at pH = 8 and gradually reduced to zero as the pH of the solution continuously increased. When pH levels were above 7, CN^−^ was stabilized and adsorbed with Co^2+^ on the surface of materials. However, the OH^−^ concentration showed another form of competition with CN^−^ for active sites when the pH was above 8, and led to the decline of adsorption. When the pH exceeds 11, an abundance of OH^−^ leads to a potential negative overall surface charge of the adsorbent, resulting in electrostatic repulsion with CN^−^ and negligible adsorption. Hence, the adsorption mechanism of Co^2+^@M-MSNs for CN^−^ predominantly involved electrostatic interactions, and the optimal pH was 8.

### 3.2. Adsorption kinetics

**Figure 6:**
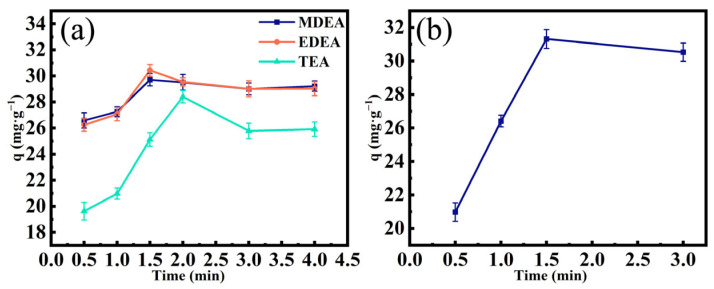
(**a**) Effect of time on the adsorption of EAs; (**b**) Effect of time on the adsorption of cyanide.

The content of EAs in the solution was determined by GC-MS [4]. The adsorption kinetics for the different targets reflected the rate at which adsorption reached equilibrium. Different times of adsorption via poly(4-VB)@M-MSNs and Co^2+^@M-MSNs were investigated. For the adsorption of EAs, the time was optimized from 30 to 240 s. As depicted in Figure 6a, the adsorption of poly(4-VB)@M-MSNs on MDEA, EDEA, and TEA exhibited a gradual rise with increasing duration. Upon reaching 90 s, the process for adsorption of MDEA and EDEA was essentially complete and reached equilibrium. It was noticed that there was a decline in adsorption with the extension of the time, which might be due to target desorption. Conversely, the adsorption for TEA reached equilibrium at 120 s. The results might be due to the complex molecular structure of TEA compared to MDEA or EDEA and the higher steric hindrance.

For the adsorption of cyanide (shown in Figure 6b), 30, 60, 90, and 180 s were investigated with the pH = 8. Notably, Co^2+^@M-MSNs quickly adsorbed cyanide within the initial 90 s, with equilibrium absorption capacity reached at 31.48 mg·g^−1^. The results were two orders of magnitude higher than the adsorption capacity previously reported in the literature [40]. The abundant positively charged binding sites on the adsorbent surface during the early period could facilitate rapid electronic adsorption. Subsequently, with the surface positive charge occupied by CN^−^, the adsorption rate was reduced until equilibrium was achieved.

To further investigate the kinetic mechanism of the adsorption process, the experimental data of EAs and cyanide were fitted with the pseudo-first-order model (PFO) and the pseudo-second-order model (PSO), respectively, and the corresponding equations were shown in (1) and (2) below:

(1) $$\log\left(q_{e}-q_{t}\right)=logq_{e}-\frac{k_{1}t}{2.303}$$

(2) $$\frac{t}{q_{t}}=\frac{1}{k_{2}{q_{e}}^{2}}+\frac{1}{q_{e}}t$$

where *q_e_* (mg·g^−1^) represented the adsorbed amount at adsorption equilibrium, *q_t_* (mg·g^−1^) represented the adsorbed amount at time *t*, *k*_1_ (min^−1^) represented the quasi-primary kinetic constant, and *k*_2_ (g·mg^−1^·min^−1^) represented the quasi-secondary kinetic constant.

The fitting results for the kinetics of EAs and cyanide are shown in Table 1. The PSO correlation coefficients (R^2^) of MDEA, EDEA, and TEA were 0.9992, 0.9985, and 0.9904, respectively, which were significantly higher than those of PFO. The results confirmed that the quasi-secondary kinetic model was better able to describe the adsorption process of poly(4-VB)@M-MSNs on the EAs. The PFO correlation coefficient of 0.9932 for cyanide was larger than that of 0.9879 for PSO, indicating that the quasi-secondary kinetic model was more suitable for describing the adsorption process of Co^2+^@M-MSNs on cyanide.

### 3.3. Adsorption Isotherm Experiment

**Figure 7:**
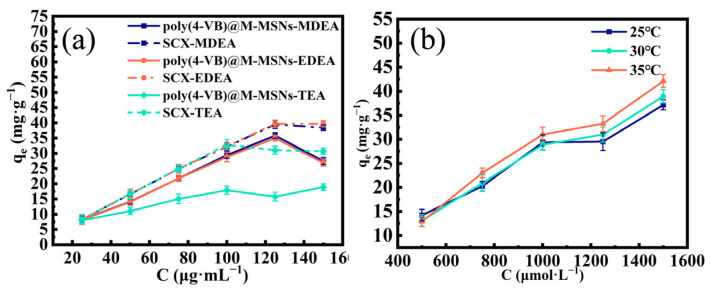
(**a**) Adsorption capacity of poly(4-VB)@M-MSNs and SCX column for different concentrations of EAs; (**b**) Adsorption at different temperatures and cyanide concentrations.

To interpret the adsorption behaviors of poly(4-VB)@M-MSNs on EAs and Co^2+^@M-MSNs on cyanide, adsorption isothermal experiments were fully carried out. Initially, the adsorption of poly(4-VB)@M-MSNs to EAs exhibited a significant increase as the initial concentrations increased, with a controlled temperature of 25 °C and adsorption duration of 90 s (seen in Figure 7a). Subsequently, adsorption isothermal experiments were utilized to evaluate the adsorption of poly(4-VB)@M-MSNs on EAs and Co^2+^@M-MSNs on cyanide. The adsorption capacities for MDEA and EDEA were observed with spiked concentration of 125 μg·mL^−1^ were 35.83 mg·g^−1^ and 35.00 mg·g^−1^ respectively. Remarkably, the adsorption efficiencies for our synthesized materials consistently remained at 90% or higher compared to the commercial SCX columns. However, TEA exhibited a lower capacity of 17.90 mg·g^−1^ obtained at the spiked concentration of 100 μg·mL^−1^. The adsorption efficiency of poly(4-VB)@M-MSNs for TEA was around 60% of that achieved by SCX columns. The difference might be also attributed to the better homogeneity of the commercial SPE materials. Meanwhile, the adsorption results of cyanide by Co^2+^@M-MSNs at varied temperatures (25 °C, 30 °C, and 35 °C) are shown in Figure 7b. The trends of the three curves were similar at different temperatures, which indicated that the changes in temperature and initial concentration showed no influence on the adsorption process. The adsorption isotherms were applied to describe the interaction behavior between adsorbent and adsorbate, i.e., the Langmuir model and the Freundlich model, and the equations of both models are shown in (3) and (4):

(3) $$\frac{c_{e}}{q_{e}}=\frac{c_{e}}{q_{m}}+\frac{1}{q_{m}K_{L}}$$

(4) $$\ln q_{e}=lnK_{F}+\frac{1}{n}lnc_{e}$$

where *q_e_* was the equilibrium adsorption amount (mg·g^−1^), *c_e_* was the equilibrium concentration (mg·L^−1^), *q_m_* was the maximum adsorption amount (mg·g^−1^), *K_L_* was the constant of Langmuir (L·mg^−1^) related to the adsorption energy, *n* was the non-homogeneity factor related to the adsorption strength, and *K_F_* was the constant of Freundlich related to the adsorption capacity.

Two models—Langmuir and Freundlich—were used to fit the experimental data, and the results of the fitted parameters are shown in Table 2 and Table 3. The correlation coefficients of Langmuir for MDEA, EDEA, and TEA were closer to 1 in the fitting results for EAs, which indicated that the adsorption of poly(4-VB)@M-MSNs on EAs could be recognized as the monolayered adsorption process and that the adsorption capacity of the adsorbent was mainly limited by the volume of the pores. However, as for the adsorption of cyanide, the Freundlich model showed a better-fitted correlation coefficient, which indicated that the adsorption of cyanide by Co^2+^@M-MSNs followed a multilayer adsorption model instead of the Langmuir model. Additionally, the *K_F_* value was closely related to the adsorption efficiency of the adsorbent, which was consistent with the selected model. The value of n could be recognized as a non-homogeneous factor related to the adsorption strength. When *1/n* was less than 1, the adsorption process was easier to carry out. The results showed that *1/n* for all cyanide adsorption isotherm models were lower than 1, which indicated that the Co^2+^-modified poly(4-VB)@M-MSNs could be easily and stably bound to cyanide and had a high affinity for cyanide.

### 3.4. DFT

The adsorption mechanisms of poly(4-VB)@M-MSNs and Co^2+^@M-MSNs on EAs and cyanide were calculated through density functional theory (DFT) calculations. The affinities of -COOH and -SO_3_H moieties for EAs were determined, respectively. As shown in Table 4, the results indicated that the energy of the products generated by the combination of -SO_3_H with MDEA, EDEA, and TEA were significantly lower than that of -COOH, indicating that poly(4-VB)@M-MSNs could more easily adsorb EAs. Meanwhile, compared with -COOH, -SO_3_H could form more covalent bonds and lead to the binding of EAs on the material surface more stable and conducive to adsorption. Similarly, the adsorption energy (Ev) results of different metal ions combined with cyanide are shown in Table 5. Compared to other metal ions, the adsorption energy of Co^2+^ combined with cyanide was the lowest, indicating that the chelate was more stable. This also proved that Co^2+^ was more suitable for preparing cationic magnetic mesoporous silicon materials for the adsorption and removal of cyanide.

### 3.5. Matrix Effect

The matrix effect (ME) can evaluate the influence of the non-target compound composition in the sample on the response value of the target compound. In our research, the ME for the EAs adsorption in the urine samples by poly(4-VB)@M-MSNs was investigated. The value of ME could be calculated as the formula: ME = [peak area of the target in the matrix sample—peak area in the pure solvent]/peak area in the pure solvent. The magnitude of ME was categorized as slight (0~20%), moderate (20~50%), or significant (>50%). In this study, ME was assessed by spiking urine samples at 100 ng·mL^−1^, resulting in an ME of 11.84 ± 1.10%. The results indicated that the ME effect associated with this sample pretreatment process and analytical method established herein was minimal.

### 3.6. Repeatability

**Figure 8:**
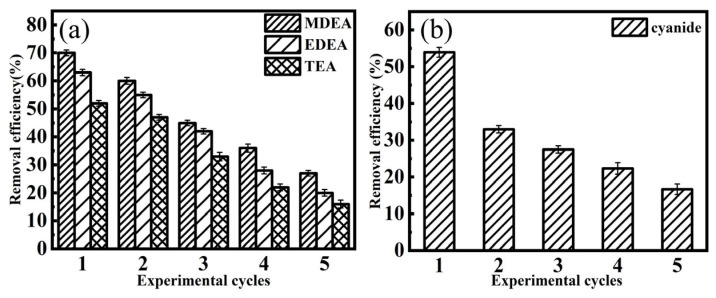
(**a**) Removal of EAs at different adsorption-desorption times; (**b**) Removal of cyanide at different adsorption-desorption times.

To assess the reusability of the materials, adsorption-desorption experiments were conducted with poly(4-VB)@M-MSNs and Co^2+^@M-MSNs, respectively. A 30% ammonia/methanol solution was applied as the desorption reagent for EAs adsorbed by poly(4-VB)@M-MSNs, while a molar ratio of hydroxocobalamin solution to cyanide of 5:1 was employed for cyanide desorption. The materials were fully washed with deionized water and dried after every adsorption-desorption cycle. The result is shown in Figure 8a,b. The adsorption capacity of poly(4-VB)@M-MSNs for EAs exhibited a slow decrease after each desorption and regeneration cycle. The decrease could be attributed to the potential degradation of certain functional groups within the materials by the alkaline reagent and the inadequate desorption of the targets. Fortunately, even after three adsorption-desorption cycles, the efficiency remained at approximately 70% of the original capacity. For the adsorption-desorption experiments of cyanide, the removal of cyanide by Co^2+^@M-MSNs also gradually decreased with the increase in the number of experiments. Meanwhile, the removal rate could still reach 50% of the original, indicating that the Co^2+^@M-MSN material had good stability and reusability.

### 3.7. Application to the Actual Samples

**Figure 9:**
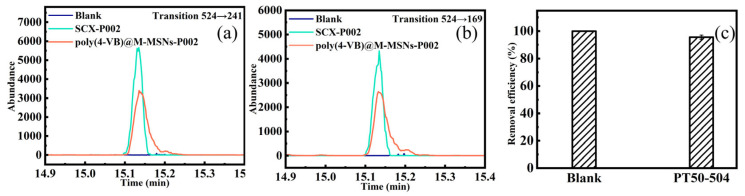
(**a**,**b**) GC-MS/MS SRM chromatogram of samples in the 6th OPCW Biomedical Proficiency Test; (**c**) UV chromatogram of samples in the 50th Official OPCW Proficiency Test.

According to the final report of the 6th Official OPCW Biomedical Proficiency Test, the P002 sample was identified as a urine sample spiked with biomarkers of mustard. The sample was pretreated with poly(4-VB)@M-MSNs, and the GC-MS/MS method was applied to analyze the sample. A similar sample pretreatment with the commercial SCX column was also carried out to compare the enrichment efficiency. The results are shown in Figure 9a,b. The blank sample showed no responses. Both the synthetic materials and the SCX column could detect the target, as the obvious peak of *m*/*z* = 524→169 and *m*/*z* = 524→241 under scanning conditions. The ratio of the two ion pairs of the target detected by the SCX column and the synthesized material was consistent, which confirmed that the detection result was correct. Through the ratio of ion pairs and peak retention times, the compound in the urine sample was unambiguously identified as TEA for the biomarkers of nitrogen mustard. The adsorption capacity of TEA by the magnetic nanomaterials reached 61.15% of that of the commercial SCX column, which demonstrated the potential of the synthetic materials for application in actual biological samples.

The water sample designated as 504 from the 50th Official OPCW Proficiency Test was utilized to evaluate the adsorption of cyanide by Co^2+^@M-MSNs. Due to the absence of target in the actual sample, cyanide was spiked in the 504 sample with the final concentration of 1.0 mmmol·L^−1^. A standard cyanide solution with the same concentration and a blank sample was also applied for contrast. The results are shown in Figure 9c. The removal percentage in the actual sample could reach 95.59% compared to the standard sample (Figure S5), indicating that Co^2+^@M-MSNs could be effectively used for the removal of cyanide in real environmental samples.

## 4. Materials and Methods

### 4.1. Materials and Instruments

#### 4.1.1. Reagents and Instruments

FeCl_3_·6H_2_O, FeCl_2_·4H_2_O, NH_3_⋅H_2_O, and (heptafluorobutyryl) imidazole (HBFI) were procured from Bai Ling Wei Technology (China). Tetraethyl orthosilicate (TEOS), cetyltrimethylammonium bromide template (CTAB), γ-methacryloxypropyl trimethoxy silane (KH-570), triethylamine, 4-vinyl benzene sulfonic acid (4-VB), and 2,2′-Azobis(2-methylpropionitrile) (AIBN) were sourced from Aladdin Reagent (Shanghai, China). CH_3_COONa, Na_2_SO_4_, and HCl were obtained from Sinopharm Chemical Reagent (Beijing, China). 1-(2-Pyridinylazo)-2-naphthol (PAN) was acquired from Macklin Reagent (China). CoCl_2_ was procured from Sigma–Aldrich (Shanghai, China). All the reagents were of analytical grade. Deionized water was sourced from Watsons Water. CH_3_OH, CH_3_CH_2_OH, isopropanol, dichloromethane, and heptane with chromatographic grade were purchased from Thermo Fisher (Waltham, MA, USA). Ammonium nitrate (1.0 mol·L^−1^) was obtained from Xiamen Haibiao Technology (Xiamen, China). N-ethyl diethanolamine (EDEA), N-methyl diethanolamine (MDEA), and triethanolamine (TEA) were synthesized in-house. KCN was sourced from Aladdin Reagent (Shanghai, China). 

The analytical instruments utilized in the research included a Trace GC Ultra gas chromatograph–Thermo DSQ II mass spectrometry system combined with a Thermo Scientific TSQ Quantum XLS triple quadrupole mass spectrometry system, an Apreo 2C field emission scanning electron microscope (Thermo Scientific, USA), a Malvern Zetasizer Nano ZS90 Nano Particle Size Potentiostat (Malvern Instruments, Malvern, UK), a CP124S Analytical Electronic Balance (Sartorius, Göttingen, Germany), a DZF-6090 Vacuum Drying Oven (Shanghai, China), a VORTEX-GENIE2 from Vortex Mixing and Equalizing Instrument Scientific Industries (Concentrator plus, Hamburg, Germany), a KQ3200E ultrasonic cleaner (Suzhou, China), a PhysiMaster 72 nitrogen adsorption instrument (Beijing, China), an Invenio-Hyperion Fourier Transform Infrared Spectrometer (Bruker, Ettlingen, Germany), an F200xField Emission Transmission Electron Microscope (FEI, Morristown, NJ, USA), and a 760CRT Spectrophotometer (Shanghai, China).

#### 4.1.2. Instrument Conditions

The EA samples treated with the anionic magnetic mesoporous nanomaterials were analyzed via gas chromatography-mass spectrometry–electron ionization/selective ion monitoring (GC-MS-EI/SIM) and gas chromatography-mass spectrometry–chemical ionization/selected reaction monitoring (GC-MS-CI/SRM) methodologies. The DB-5MS column (5%-phenyl-95%-methypolysiloxane, 30 m × 0.25 mm × 0.25 µm) with the helium as the carrier gas with a flow rate of 1.0 mL·min^−1^ was employed for the chromatographic separation. The temperature of the injector was 250 °C. The initial column temperature was settled at 50 °C, followed by the programmed ramp of 10 °C·min^−1^ until 280 °C, which was held for 5.0 min. The solvent delay was 5.0 min, and the ionization energy was 70 ev. Scanning parameters included a scanning time of 0.05 s and a width of 0.002 s. The ion source and transmission line temperatures were set at 250 °C and 280 °C, respectively. Selected ions were applied as *m*/*z* = 169 and *m*/*z* = 241 for both MDEA and TEA derivatives, and *m*/*z* = 298 and *m*/*z* = 169 for EDEA derivatives. The column and instrumental conditions for GC-MS-CI/SRM analysis remained consistent with previously reported parameters [4].

A spectrophotometer was employed for the analysis of cyanide samples treated with cationic magnetic mesoporous nanomaterials. Absorbance measurements were performed with a rapid scanning model, and the sampling interval was 1 nm. The scan wavelength ranged from 200 nm to 800 nm, with a vertical coordinate range set at 0–0.5 A. Tungsten served as the lamp source, with a switching wavelength of 330 nm and a slit width of 2 nm. Each sample was measured three times and averaged to eliminate errors.

### 4.2. Preparation of Ionic Magnetic Mesoporous Materials

#### 4.2.1. Synthesis of Magnetic Mesoporous Silicon Nanoparticles (M-MSNs)

Initially, Fe_3_O_4_ magnetic nanoparticles (MNPs) were synthesized via the co-precipitation method by FeCl_3_·6H_2_O, FeCl_2_·4H_2_O and NH_3_⋅H_2_O [50,51]. Subsequently, the MNPs (3.0 g) were dispersed in a solution consisting of 160 mL of ethanol, 40 mL of deionized water, and 2 mL of ammonia (25%) with sufficient ultrasonic oscillation. A volume of 1.2 mL of tetraethyl orthosilicate (TEOS) was slowly dripped into the solution under nitrogen protection and stirred at room temperature for 6 h. The reaction products were washed with ethanol and deionized water until the pH returned to neutral. The materials were dried at 60 °C by overnight and the magnetic silicon nanoparticles (MSNs) were obtained. The MSNs were then dispersed in a solution consisting of 100 mL of ethanol, 100 mL of deionized water, and 1.5 mL of ammonia (NH_3_ 25%). 2.0 g of cetyltrimethylammonium bromide (CTAB) and 3.0 mL of TEOS were added and stirred at room temperature for 6 h. The reaction products were sufficiently washed with deionized water and ethanol. Finally, the resultant product was redispersed in the 30 mg·mL^−1^ ammonium nitrate/ethanol solution at 80 °C for 12 h to ensure the complete removal of CTAB. The above cleaning steps were repeated, and the magnetic mesoporous silicon nanoparticles (M-MSNs) were obtained.

#### 4.2.2. Synthesis of Poly(4-VB)@M-MSNs and Co^2+^@M-MSNs

KH-570 was utilized for the modification of the double bond. A mass of 1.0 g of M-MSNs was dispersed in 200 mL of isopropanol and then transferred to a three-necked flask. A volume of 1.0 mL of triethylamine was added and the mixture was thoroughly stirred subsequently. KH-570 (6.0 mL) and ethanol (12.0 mL) were slowly dripped under the nitrogen protection and the reaction system was maintained at 70 °C for 12 h. The final product KH-570@M-MSNs was collected via magnetic separation and washed to neutral with water and ethanol.

Next, KH-570@M-MSNs were re-dissolved in 100 mL of isopropanol and combined with 100 mL of acetonitrile solution containing 0.15 g of 2,2′-azobis(2-methylpropionitrile) (AIBN) in the three-necked flask. Under the nitrogen protection, 3.0 g of 4-vinylbenzenesulfonic acid (4-VB) was added and the reaction mixture was mechanically stirred at 80 °C/300 rpm for 16 h. The anionic magnetic mesoporous nanomaterials of poly(4-VB)@M-MSNs were successfully collected via magnetic separation. The materials were washed with deionized water and ethanol and dried at 60 °C overnight.

Finally, 3.0 mg of poly(4-VB)@M-MSNs were accurately weighed and transferred into the 5.0 mL centrifuge tube, to which 3.0 mL of 1 mmol·L^−1^ CoCl_2_ solution was added. The mixture was subjected to shaking at 1000 rpm at room temperature for 5 h. The materials were followed by magnetic separation and 2–3 cycles of washing with deionized water. The cationic magnetic mesoporous nanomaterials, Co^2+^@M-MSNs, were synthesized.

### 4.3. Sample Preparation

#### 4.3.1. Preparation of EA Samples

The poly(4-VB)@M-MSNs were employed for the enrichment and purification of EAs (MDEA, EDEA, and TEA) in different matrixes. Specifically, 3.0 mg of poly(4-VB)@M-MSNs was accurately weighed into a centrifuge tube with 1.0 mL of an EAs spiked sample. The centrifuge tube was maintained at room temperature and shaken at 1000 rpm to evaluate the impact of multiple contact times on the adsorption efficiency. After the adsorption process, the centrifuge tube was subjected to a magnetic rack, and the supernatant was separated, which was concentrated to dryness at 45 °C and 1600 r·min^−1^ by a vacuum centrifugal concentrator. The resultant solution was then redissolved with 0.5 mL of acetonitrile. The sample was transferred to a 4.0 mL sample vial and 50 μL of (heptafluorobutyryl) imidazole (HBFI) was added. The derivatization reaction was maintained at 50 °C for 8 min. After the heating incubation, 0.5 mL of n-heptane was introduced to extract the derivative from the mixture. The final extraction solution was transferred to a 1.5 mL sample vial and concentrated to 0.1 mL with mild nitrogen. Finally, the sample was analyzed by GC-MS to determine the concentration of the EAs.

#### 4.3.2. Preparation of Cyanide Samples

The adsorption of cyanide from environmental samples was accomplished by Co^2+^@M-MSNs. A mass of 3.0 mg of Co^2+^@M-MSNs was accurately weighed into a 5.0 mL centrifuge tube, followed by the addition of 3.0 mL of a cyanide solution with appropriate concentration. The adsorption systems were maintained at 30 °C and 1000 rpm for 5 min. After the adsorption process, the Co^2+^@M-MSNs were isolated by magnetic separation and the supernatant was transferred to a 4.0 mL sample tube. A volume of 1.0 mL of the supernatant was mixed with 2.0 mL of 2.0 mmol·L^−1^ CoCl_2_ solution and shaken at 1000 rpm for 10 min at room temperature. The absorbance of the reacting sample was measured at 309 nm using a UV spectrophotometer.

### 4.4. Characterization

Scanning electron microscopy (SEM) and transmission electron microscopy (TEM) and compositions of Fe_3_O_4_, MSNs, M-MSNs, and poly(4-VB)@M-MSNs were identified by X-ray diffraction (XRD) analysis. Further interpretation of the chemical states of the elements was obtained via X-ray photoelectron spectroscopy (XPS). The saturation magnetization strength and superparamagnetic properties of the prepared Fe_3_O_4_ and M-MSNs were measured by the vibrating sample magnetometer (VSM) needle, whose magnetic field strength was set at −20.0000e–20.0000e. The specific surface area of the samples was calculated by the Brunauer–Emmet–Teller (BET) model, which was completed by the nitrogen adsorption and desorption tests. Furthermore, the chemical functional group composition and structure of Fe_3_O_4_, MSNs, CTAB@MSNs, and M-MSNs were fully investigated by the Fourier-transform infrared spectrometer (FT-IR). At last, the surface potentials of the nanomaterials were determined utilizing a nanoparticle size potentiostat (zeta potential) to confirm the presence of -SO_3_H and Co^2+^.

### 4.5. Adsorption Isotherm

To investigate the maximum adsorption capacity and adsorption mechanism between the nanomaterials and the targets, the isothermal adsorption experiments were carried out. A total of 3.0 mg poly(4-VB)@M-MSNs were dispersed in the aqueous solutions of EAs spiked as the concentration of 25, 50, 75, 100, 125, and 150 μg·mL^−1^, respectively. The adsorption was maintained for 3 min with shaking at 1000 rpm. The material was isolated by magnetic separation. The supernatant was combined and the concentration of EAs was determined by GC-MS.

For the cyanide isothermal adsorption experiments, 3.0 mg Co^2+^@M-MSNs were dispersed in the aqueous solutions of spiked cyanide as the concentration of 500, 750, 1000, 1250, and 2000 μmol·L^−1^ at 25 °C, 30 °C and 35 °C, respectively. The adsorption was maintained for 5 min with shaking at 1000 rpm. The material was isolated by magnetic separation. The supernatant was combined and the concentration of cyanide was determined by UV spectrophotometer at 309 nm. The equilibrium adsorption capacity *q_e_* (mg·g^−1^) of EAs and cyanide was measured via the following equation:

(5) $$q_{e}=\frac{{(c}_{0}-c_{e})\times V}{m}$$

(6) $$q_{e}=\frac{(c_{0}-c_{e})\times V\times 10^{-3}\times M}{m}$$

where *c*_0_ (μg·mL^−1^ or μmol·L^−1^) indicated the initial concentration of EAs or cyanide, *c_e_* (μg·mL^−1^ or μmol·L^−1^) indicated the equilibrium concentration, *V* (mL) indicated the volume of EAs or cyanide, *M* (g·mol^−1^) indicated the molar mass of KCN, and *m* (mg) indicated the dosage of adsorbent.

### 4.6. Adsorption Kinetics

To investigate the adsorption kinetics of EAs, 3.0 mg poly(4-VB)@M-MSNs was dispersed in 1.0 mL EA standard solution (10 μg·mL^−1^). The concentration of the remaining EAs in the supernatant was determined by GC-MS at certain time intervals (30, 60, 90, 120, 180, and 240 s).

The adsorption kinetics of cyanide was revealed with 3.0 mg of Co^2+^@M-MSNs dispersed in 3.0 mL of cyanide standard solution (1000 μmol·L^−1^). The concentration of remaining cyanide in the supernatant was calculated by the absorbance at 309 nm for 30, 60, 90, and 180 s. The adsorption amounts *q_t_* (mg·g^−1^) of EAs and cyanide at different contact times were calculated as follows:

(7) $$q_{t}=\frac{{(c}_{0}-c_{t})\times V}{m}$$

(8) $$q_{e}=\frac{(c_{0}-c_{t})\times V\times 10^{-3}\times M}{m}$$

where *c*_0_ (μg·mL^−1^ or μmol·L^−1^) indicated the initial concentration of EAs or cyanide, *c_t_* (μg·mL^−1^ or μmol·L^−1^) indicated the concentration of EAs or cyanide at the moment *t*, *V* (mL) indicated the volume of EAs or cyanide, *M* (g·mol^−1^) indicated the molar mass of KCN, and *m* (mg) indicated the dosage of adsorbent.

### 4.7. DFT Calculations

DFT calculations were applied to evaluate whether the selection of modification was reasonable. All DFT calculations were performed via the DMOL3 module of the Materials Studio 2021 software. Calculations by the local density approximation (LDA) underestimated the equilibrium distances and overestimated the bond energies. Therefore, the structure optimization and energy calculations for the most stable geometries in this study were based on generalized gradient approximation (GGA) functions with PBE corrections. In our research, the DFT-D correction proposed by Grimme was applied to explain the weak van der Waals interactions.

## 5. Conclusions

In our research, two types of ionic magnetic nanoparticles (poly(4-VB)@M-MSNs and Co^2+^@M-MSNs) with good magnetization intensity, large specific surface area, and high ability to selectively adsorb EAs and cyanide were successfully synthesized. The results of adsorption kinetics showed that the adsorption for the two targets could reach equilibrium within 90 s. Compared to the commercial SCX column, the poly(4-VB)@M-MSNs achieved good recovery performance for EAs, and the adsorption efficiencies of MDEA and EDEA could be maintained at 90%. Meanwhile, materials such as Co^2+^@M-MSNs also achieved highly effective removal of cyanide, and the adsorption capacity could reach up to 31.48 mg·g^−1^ within 90 s, much higher than the 0.14 mg·g^−1^ reported in the literature [40]. Both the two materials showed good reproducibility after three cycles of adsorption and the adsorption or removal efficiencies still maintained more than 70% and 50% of the original, respectively. Notably, the materials showed good stability and resistance to matrix interference in the complex bio-samples. The materials could also be successfully applied to the detection of actual samples from the OPCW Proficiency Test, which showed excellent application potential in the verification of CWAs and the decontamination of toxic substances.

## Figures and Tables

**Table 1:** Fitting parameters of adsorption kinetic models.

		PFO			PSO	
	R^2^	*k*_1_ (min^−1^)	*q_e_* (mg·g^−1^)	R^2^	*k*_2_ (g·mg^−1^·min^−1^)	*q_e_* (mg·g^−1^)
MDEA	2.590 × 10^−3^	0.1652	0.1270	0.9992	0.9967	29.41
EDEA	1.105 × 10^−2^	0.4028	0.1118	0.9985	1.089	29.43
TEA	0.7807	0.3622	8.770	0.9904	0.2124	27.49
KCN	0.9932	−0.9044	14.64	0.9879	1.086 × 10^−2^	66.05

**Table 2:** Fitting parameters for the EA adsorption isotherm model.

	Langmuir Equation			Freundlich Equation		
	R^2^	*K_L_* (L·mg^−1^)	*q_m_* (mg·g^−1^)	R^2^	*K_F_*	1·n^−1^
MDEA	0.9768	0.6226	28.47	0.7399	1.068 × 10^−3^	2.950
EDEA	0.9761	0.5917	27.99	0.7549	3.088 × 10^−3^	2.666
TEA	0.9705	0.1591	18.84	0.8943	7.976 × 10^−5^	4.789

**Table 3:** Fitting parameters for the cyanide adsorption isotherm model.

Temperature/°C	Langmuir Equation			Freundlich Equation		
	R^2^	*K_L_* (L·mg^−1^)	*q_m_* (mg·g^−1^)	R^2^	*K_F_*	1·n^−1^
25	0.2404	1.251 × 10^−2^	85.91	0.6940	2.168	0.8273
30	3.334 × 10^−2^	4.668 × 10^−3^	154.80	0.8726	2.122	0.6724
35	3.000 × 10^−3^	1.180 × 10^−3^	613.50	0.8231	4.383	0.7289

**Table 4:** Adsorption energy (Ev) of -COOH and -SO_3_H and with EAs.

Binding Product	Ev (kJ·mol^−1^)
MDEA-COOH	−592.10
MDEA-SO_3_H	−1028.68
EDEA-COOH	−632.22
EDEA-SO_3_H	−1067.96
TEA-COOH	−707.60
TEA-SO_3_H	−1143.28

**Table 5:** Adsorption energy (Ev) of different metal ions with CN^−^.

Metal Ions	Ev (kJ·mol^−1^)
Cu	28.49
Ca	−18.20
Fe	−25.63
Mg	−22.46
Co	−27.31

## Data Availability

Data sharing not applicable to this article as no datasets were generated or analyzed during the current study.

## Supplementary Materials

The following supporting information can be downloaded at: https://www.mdpi.com/article/10.3390/ijms25126470/s1.
