# Supplementary material for: Highly Efficient Separation of Ethanol Amines and Cyanides via Ionic Magnetic Mesoporous Nanomaterials
Source: Int J Mol Sci. 2024 Jun 12;25(12):6470. doi: 10.3390/ijms25126470 (PMC11203903; doi:10.3390/ijms25126470)
Supplement: Supplementary file 1 [file ijms-25-06470-s001.zip › ijms-2999326-supplementary.pdf]

Catalog for supplementary material

1. Table S1. Abbreviations and acronyms S-2
2. Calculation of average crystallite size... S-3
3. BET and pore size distribution of materials at different concentrations and temperatures....... S-4
4. The XPS spectra of C1s before and after of Fe_3O_4@SiO_2 modification of KH-570 .... S-5
5. UV spectrogram of cyanide absorption.. S-6
6. Zeta potential of different synthesis steps S-7
7. XPS of different synthesis steps... S-8
8. C% of synthetic materials in different steps.. S-9

1. Table S1. Abbreviations and acronyms

Abbreviation	Full name
AIBN	2,2'-Azobis(2-methyl propionitrile)
BET	Brunauer-Emmet-Teller
BSTFA	N, O-bis(trimethylsilyl trifluoroacetamide)
CTAB	cetyltrimethylammonium bromide
CWAs	chemical warfare agents
CWC	Chemical Weapons Convention
DFT	density functional theory
EAs	ethanol amines
EDEA	N-ethyl diethanolamine
FT-IR	Fourier-transform infrared spectrometry
GC-MS	gas chromatography-mass spectrometry
GC-MS-CI/SRM	gas chromatography-mass spectrometry chemical ionization selected reaction monitoring
GC-MS-EI/SIM	gas chromatography-mass spectrometry electron ionization selective ion monitoring
HFBI	Heptafluorobutyryl imidazole
HN1	bis(2-chloroethyl)ethylamine
HN2	bis(2-chloroethyl)methylamine
HN3	tris(2-chloroethyl)amine
KH-570	γ -methacryloxypropyl trimethoxy silane
LC-MS	liquid chromatography-mass spectrometry
LLE	liquid-liquid extraction
MDEA	N-methyl diethanolamine
ME	matrix effect
M-MSNs	magnetic mesoporous silicon nanoparticles
MNPs	magnetic nanoparticles
MSNs	magnetic silicon nanoparticles
NMs	nitrogen mustards
OPCW	Organization for the Prohibition of Chemical Weapons
PAN	1-(2-Pyridinylazo)-2-naphthol
PFO	pseudo-first-order model
PSO	pseudo-second-order model
SCX	strong cation-exchange
SEM	scanning electron microscopy
SMs	sulfur mustards
SPE	solid-phase extraction
TEA	triethanolamine
TEM	transmission electron microscopy
TEOS	Tetraethyl orthosilicate
TGA	thermogravimetric analysis
VSM	vibrating sample magnetometer
XRD	X-ray diffraction
XPS	X-ray photoelectron spectroscopy
4-VB	4-vinyl benzene sulfonic acid

2. Calculation of average crystallite size

The formula for calculating the average crystal size is Scherrer as follows:

$$D = \frac{k\lambda}{\beta cos\theta}$$

Where D is crystal size (nm); k is the Scherrer constant, k=0.89; λ denotes the X-ray wavelength, λ=0.15406nm; β denotes the FWHM half-peak width, measured in radians; θ denotes the diffraction angle.

Table S2: The calculation results of Fe_3O_4 particles

**Centroid-A**	**FWHM**	**XS**	**D（nm）**
30.248	0.812	102	10.2
35.528	0.745	113	11.3
43.202	0.620	140	14.0
53.656	0.587	124	12.4
57.272	0.723	157	15.7
62.776	0.687	137	13.7

The average particle size is 12.88 nm.

Table S3: The calculation results of Fe_3O_4@SiO_2 particles

**Centroid-A**	**FWHM**	**XS**	**D（nm）**
30.189	0.538	156	15.6
35.572	0.670	126	12.6
43.295	0.516	199	19.9
53.742	0.575	157	15.7
57.244	0.765	119	11.9
62.862	0.642	147	14.7

The average particle size is 15.06 nm.

3. BET and pore size distribution of materials at different concentrations and temperatures

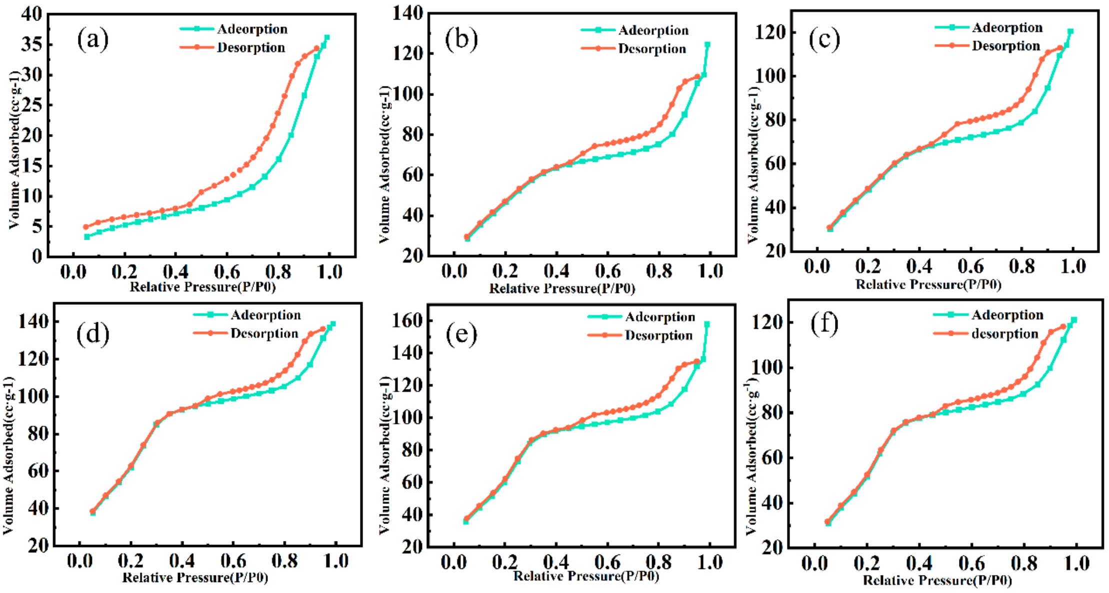

Figure S1: (a) BET of 0 mg·mL^{-1} ammonium nitrate/ethanol; (b) BET of 10 mg·mL^{-1} ammonium nitrate/ethanol; (c) BET of 20 mg·mL^{-1} ammonium nitrate/ethanol; (d) BET of 30 mg·mL^{-1} ammonium nitrate/ethanol; (e) BET of 40 mg·mL^{-1} ammonium nitrate/ethanol; (f) BET of 50 mg·mL^{-1} ammonium nitrate/ethanol.

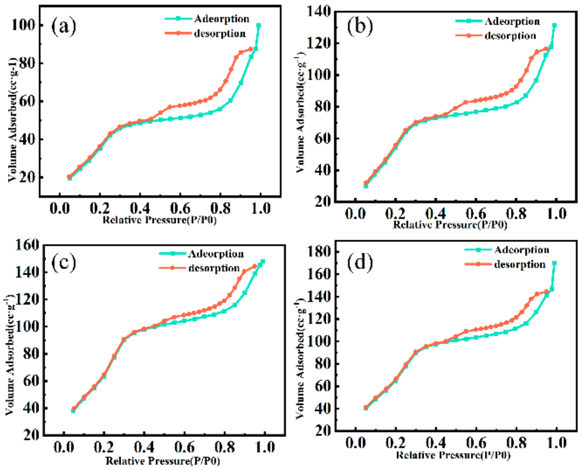

Figure S2: (a) BET at 60 °C; (b) BET at 70 °C; (c) BET at 80 °C; (d) BET at 90 °C

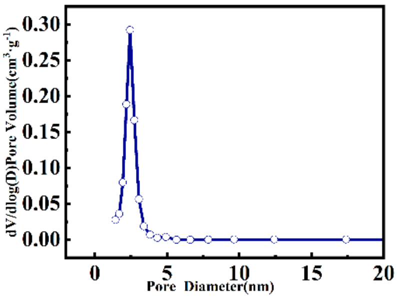

Figure S3: The pore size distribution of the material when BET is 341.7366 m^2·g^{-1}.

4. The XPS spectra of C1s before and after of $Fe_3O_4@SiO_2$ modification of KH-570

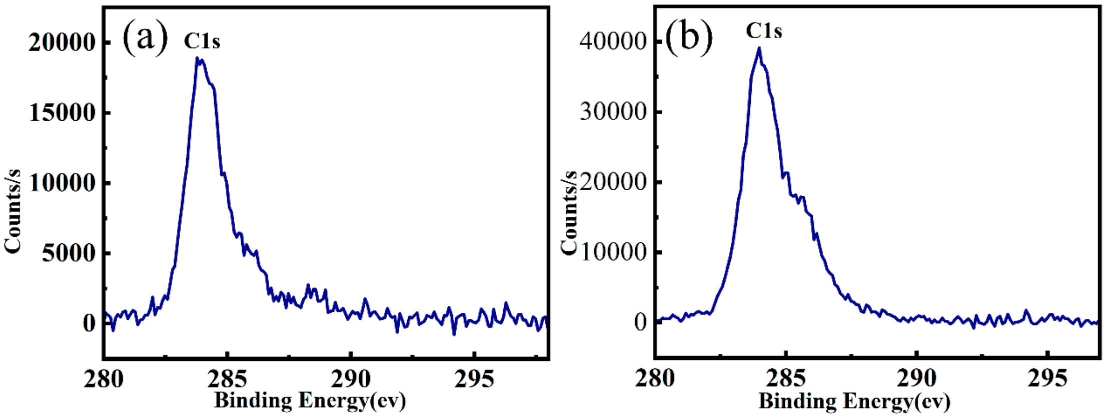

Figure S4: (a) XPS spectra of $Fe_3O_4@SiO_2$; (b) XPS spectra of $Fe_3O_4@SiO_2$-KH570

Table S4: The peak table of $Fe_3O_4@SiO_2$

Name	Peak BE	Height CPS	FWHM eV	Area (P) CPS.eV	Atomic %	Q	SF
	531.08	305115.52	4.36	7411150.03	0	0	0
Si2p	102.56	25078.46	1.9	57734.81	14.11	1	0.9
S2p	169.91	394.74	0	2164.83	0.26	1	1.881
N1s	401.18	1260.19	1.07	5204.68	0.82	1	1.676
O1s	531.97	161733.4	2.09	559646.41	56.79	1	2.881
Fe2p	710.31	63133.04	3.88	558721.9	13.28	1	14.353
Co2p	783.35	10609.21	10.99	129609.82	2.61	1	18.235
C1s	284.04	18685.85	1.74	49462.09	12.13	1	1

Table S5: The peak table of $Fe_3O_4@SiO_2$-KH570

Name	Peak BE	Height CPS	FWHM eV	Area (P) CPS.eV	Atomic %	Q	SF
	530.7	230114.64	4.46	6004453.62	0	0	0
Si2p	102.01	18920.09	2.02	46948.11	12.79	1	0.9
S2p	168.82	632.46	0.26	3893.51	0.53	1	1.881
N1s	401.34	1559.07	2.16	7982.12	1.41	1	1.676
O1s	531.58	127819.45	1.97	426996.24	48.31	1	2.881
Fe2p	709.64	39685.1	3.78	309542.78	8.2	1	14.353
Co2p	782.42	6568.05	9.77	81528.74	1.83	1	18.235
C1s	284.06	37753.11	1.86	98550.38	26.94	1	1

5. UV spectrogram of cyanide absorption

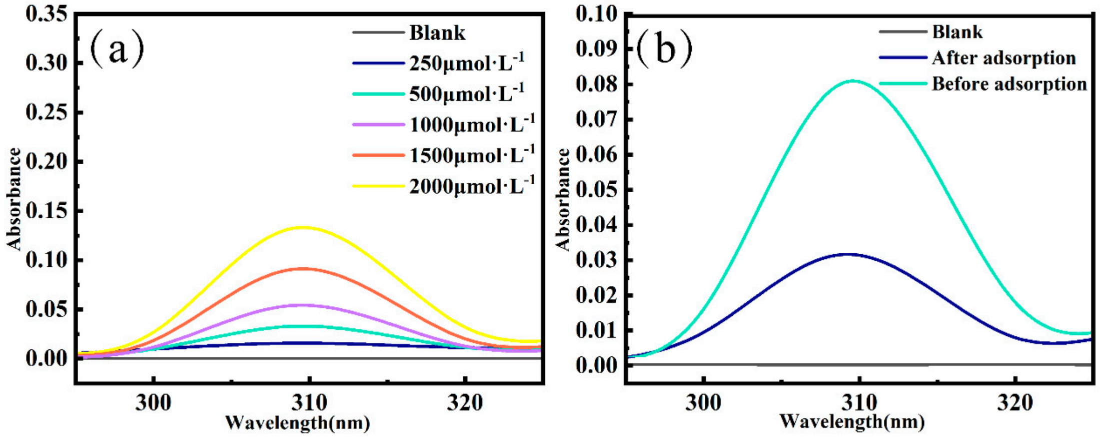

Figure S5: (a) UV-Vis spectra of magnetic nanomaterials adsorbing different cyanide compounds; (b) UV-Vis spectra of magnetic nanomaterials before and after adsorption of actual samples

6. Zeta potential of different synthesis steps

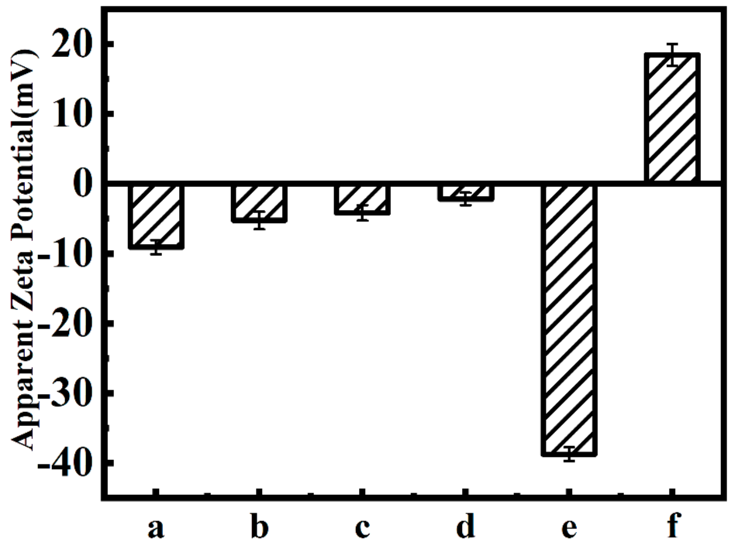

Figure S6:The Zeta potential of (a) MNPs; (b) MSNs; (c) M-MSNs; (d) KH-570@MSNs; (e) poly(4-VB)@M-MSNs; (f) Co^{2+}@M-MSNs

7. XPS of different synthesis steps

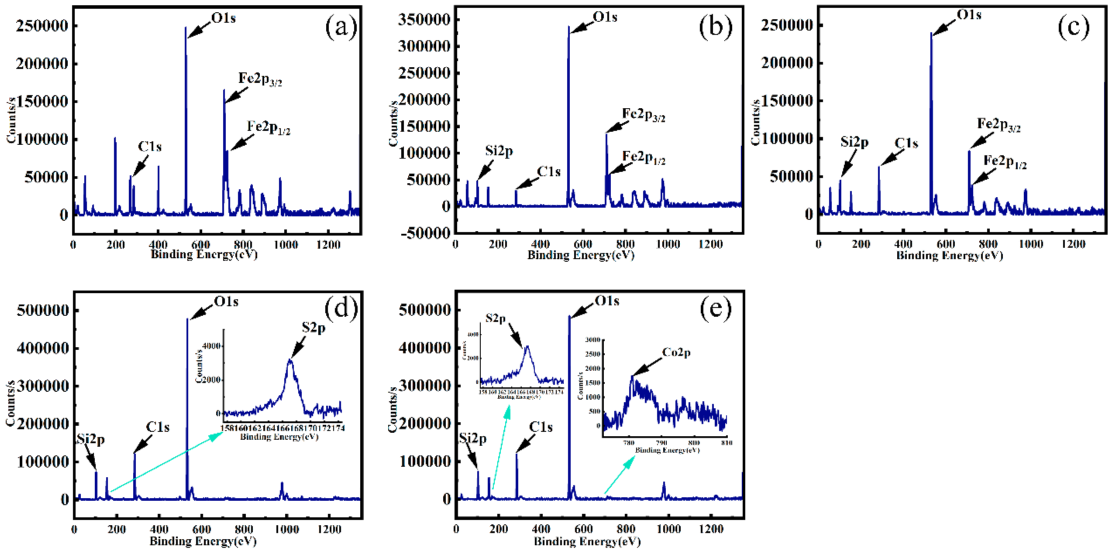

Figure S7: The XPS of (a) MNPs; (b) MSNs; (c) KH-570@MSNs; (d) poly(4-VB)@M-MSNs; (e) Co^{2+}@M-MSNs

8. C% of synthetic materials in different steps

Table S6: The C% of synthetic materials in different steps

Name	Peak BE	Height CPS	FWHM eV	Area (P) CPS.eV	Atomic %	Q	SF
Fe_3O_4	283.87	19534.21	2.06	65292.88	19.72	1	1
MSNs	284.04	18685.85	1.74	49462.09	12.13	1	1
KH-570@MSNs	284.06	37753.11	1.86	98550.38	26.94	1	1
poly(4-VB)@M-MSNs	284.1	60690.62	1.6	162068.34	34.88	1	1
Co^{2+}@M-MSNs	284.16	57772.86	2.69	161332.34	34.56	1	1
